# Esketamine versus placebo on time to remission in major depressive disorder with acute suicidality

**DOI:** 10.1186/s12888-023-05017-y

**Published:** 2023-08-11

**Authors:** Dong-Jing Fu, Qiaoyi Zhang, Ling Shi, Stephane Borentain, Shien Guo, Maju Mathews, Joana Anjo, Abigail I. Nash, Marguerite O’Hara, Carla M. Canuso

**Affiliations:** 1 Janssen Research & Development, LLC, Titusville, NJ 08560 USA; 2 Janssen Global Services, LLC, Titusville, NJ 08560 USA; 3 Evidera-PPD, Bethesda, MD 20814 USA; 4 Janssen Cilag-Farmacêutica, 2740-262 Porto Salvo, Portugal; 5 Janssen Scientific Affairs, LLC, Titusville, NJ 08560 USA

**Keywords:** Esketamine nasal spray, Depressive disorder, Suicidal ideation, Remission, Antidepressant agents

## Abstract

**Background:**

Esketamine (ESK) nasal spray, taken with oral antidepressant therapy, is approved for the treatment of depressive symptoms in adults with major depressive disorder (MDD) with acute suicidal ideation or behavior. In pooled analyses of two pivotal phase 3 studies, ASPIRE I and II, remission rates were consistently higher among patients with MDD with active suicidality who were treated with ESK + standard of care (SOC) versus placebo (PBO) + SOC at all time points in the double-blind and most time points in the follow-up phases. The current analysis of the ASPIRE data sets assessed the effect of ESK + SOC versus PBO + SOC on additional remission-related endpoints: time to achieving remission and consistent remission, proportion of patients in remission and consistent remission, and days in remission.

**Methods:**

Post hoc analysis of pooled data from ASPIRE I and II (*N* = 451). Remission and consistent remission were defined as Montgomery-Åsberg Depression Rating Scale (MADRS) total score ≤ 12 at any given visit or two consecutive visits, respectively. Combined endpoints utilizing Clinical Global Impression-Severity of Suicidality-revised version [CGI-SS-r] ≤ 1 (i.e., not suicidal/questionably suicidal) along with the remission and consistent remission definitions (i.e., MADRS total score ≤ 12) were also examined.

**Results:**

The median times to remission and consistent remission of MDD were significantly shorter in ESK + SOC versus PBO + SOC (15 versus 23 [*p* = 0.005] and 23 versus 50 days [*p* = 0.007], respectively) and a greater proportion of patients in ESK + SOC achieved remission and consistent remission by Day 25 (65.2% versus 55.5% and 54.2% versus 39.8%, respectively). Similar results were obtained using the combined endpoint for both remission definitions. The median percent of days in remission during the double-blind treatment phase was significantly greater in ESK + SOC (27.1% or 5 days) versus PBO + SOC (8.3% or 2 days; *p* = 0.006), and the significant difference was maintained during follow-up.

**Conclusion:**

Treatment with ESK + SOC versus PBO + SOC resulted in significantly shorter time to remission, greater proportion of patients in remission, and greater percent of days in remission using increasingly rigorous definitions of remission. These findings underscore the clinical benefits of ESK for adults with MDD with suicidality.

**Trial registration:**

ClinicalTrials.gov registry NCT03039192 (registered February 1, 2017) and NCT03097133 (registered March 31, 2017).

**Supplementary Information:**

The online version contains supplementary material available at 10.1186/s12888-023-05017-y.

## Introduction

Depression is a devastating psychiatric illness that is a main cause of disability worldwide [1]. In 2017, there were roughly 264 million individuals worldwide living with depression [2]. Major depressive disorder (MDD) can lead to suicide and is the most prevalent psychiatric diagnosis among those who have taken their own life [3, 4]. While the main objective of MDD treatment is remission of depressive symptoms, reduction of suicidality is also an important treatment goal.

The presence of active suicidal ideation with intent in patients with MDD constitutes a psychiatric emergency requiring urgent treatment of the underlying disease (ie, MDD). Current standard practice for these patients frequently includes hospitalization to ensure close monitoring along with treatment of symptoms of MDD with an antidepressant medication and development of a comprehensive crisis management plan before discharge from inpatient care [5]. Hospitalization, however, if accessible or acceptable by patients, is generally temporary, and patients remain at high risk of rehospitalization [6] and death by suicide [7, 8] after discharge. Indeed, American Psychiatric Association guidelines describe hospitalization not as a treatment for suicidality by itself, but rather a setting from which evaluation and treatment can be facilitated [9]. While standard antidepressants are often effective in treating severe depressive symptomatology, they typically take 4–6 weeks to achieve full clinical efficacy, which limits their utility in crisis situations and creates a treatment gap for patients in need of urgent symptom control. Patients thus remain vulnerable early in the course of treatment [10]. These data underscore the need for fast-acting treatment options that can reduce depressive symptoms rapidly in a psychiatric emergency.

In 2020, the U.S. Food and Drug Administration approved SPRAVATO® (esketamine [ESK] nasal spray), taken with an oral antidepressant, for the treatment of depressive symptoms in adults with MDD with acute suicidal ideation or behavior [11]. A similar indication has recently been approved in Europe [12]. ESK, the S-enantiomer of ketamine and an N-methyl-D-aspartate receptor antagonist, acts through a primary mechanism that differs from that of traditional monoaminergic antidepressants [13, 14]. It has a 4-week treatment regimen and is the first approved medication that significantly reduces depressive symptoms within 24 h. Thus, it provides a novel treatment option to quickly improve symptoms while a longer-term, comprehensive care plan can be established and an oral antidepressant can exert full effect [15]. ESK nasal spray approval in this indication was based on two phase 3, double-blind, multicenter registration trials in patients with MDD and active suicidal ideation with intent, ASPIRE I [16] and ASPIRE II [17]. In both trials, patients who received ESK nasal spray, given in addition to comprehensive standard of care (SOC) treatment (ESK + SOC), exhibited a significantly greater reduction in depressive symptoms than those who received placebo (PBO) nasal spray plus SOC (PBO + SOC) [18]. This treatment effect was observed as early as 4 h after the first dose of ESK and generally remained throughout the 4-week double-blind treatment phase. In the ASPIRE studies, severity of suicidality was improved rapidly in both ESK + SOC and PBO + SOC arms, but the treatment difference on this endpoint was not statistically significant 24 h after the first dose. This may be due in part to the non-specific clinical benefit from hospitalization and high clinical contact with study participants or the method used to evaluate rapid change in suicidality.

Among patients with MDD and suicidal ideation or behavior, the incidence of suicide attempts during a major depressive episode is 21-fold higher than during remission and the length of time spent in a major depressive episode is a risk factor for suicide attempts [19]. This suggests that remission of MDD should be an urgent treatment goal for patients with acute suicidality. This objective is further supported by American Psychiatric Association practice guidelines for patients with suicidal behaviors that identify treatment of the underlying illness as a main goal [9]. In the pooled analyses of ASPIRE trials, remission rates were consistently higher among patients treated with ESK + SOC at all time points in the double-blind and most time points in the follow-up phases, but time to remission and time spent in remission as clinical endpoints have not yet been fully explored to assess the effect of ESK in the ASPIRE trials. Furthermore, the consistency of remission, which is critical to provide relief to patients, has not been evaluated in the ASPIRE data sets.

### Aims of the study

This post hoc analysis assessed the effect of ESK + SOC versus PBO + SOC on time to achieving remission and consistent remission (as assessed by the Montgomery-Åsberg Depression Rating Scale [MADRS] total score alone or combined with an assessment of suicidality by the Clinical Global Impression-Severity of Suicidality-revised version [CGI-SS-r] scale) as well as time spent in remission, using pooled data from two pivotal phase 3 trials, ASPIRE I and ASPIRE II.

## Methods

### Patients

This post hoc analysis used data pooled from two identically designed, double-blind, randomized, placebo-controlled, multicenter, phase 3 studies: ASPIRE I (NCT03039192) and ASPIRE II (NCT03097133) [16, 17]. In both studies, eligible patients (aged 18–64 years of age) met the Diagnostic and Statistical Manual of Mental Disorders, Fifth Edition (DSM-5) [20] criteria for MDD without psychotic features as assessed by the Mini International Neuropsychiatric Interview [21]. Inclusion criteria included moderate to severe MDD (MADRS total score > 28), current suicidal ideation with intent in the past 24 h, and clinically warranted psychiatric hospitalization. Full inclusion and exclusion criteria have been previously published [16, 17].

### Study design

**Fig. 1:**
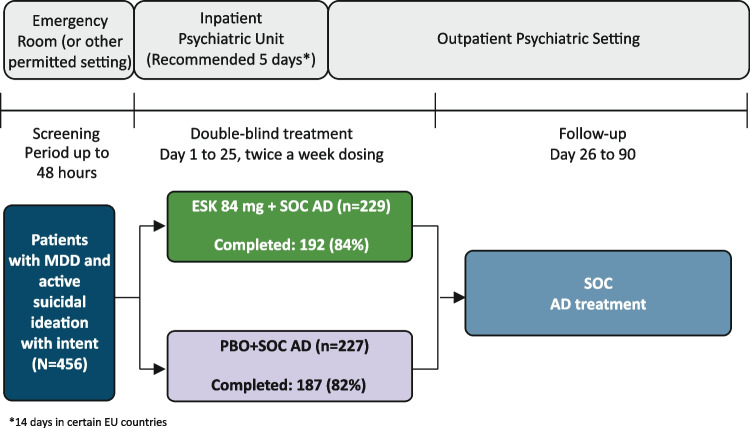
Study design of ASPIRE I and ASPIRE II. Two patients in each treatment group were excluded from these analyses because they did not receive a dose of intranasal study drug after randomization. Changes in MADRS total score and CGI-SS-r were assessed at 4 and 24 h post first dose, twice per week pre-dose during the double-blind phase, and at varied time intervals during the follow-up phase (days 28–39: twice weekly; days 46–53: weekly; days 67–90: biweekly). AD, antidepressant; ESK, esketamine; MDD, major depressive disorder; PBO, placebo; SOC, standard of care

ASPIRE I was conducted from June 2017 to December 2018 in the United States, Europe, Asia, and South Africa. ASPIRE II was conducted from June 2017 to April 2019 in North America, South America, and Europe. Both studies consisted of three phases: (1) a 24- to 48-h screening phase to assess patients’ eligibility for study enrollment, (2) a 4-week double-blind treatment phase (days 1–25), and (3) a 9-week post treatment follow-up phase (days 26–90). During the 4-week double-blind treatment phase, patients were randomly assigned (1:1) to receive either ESK 84 mg or PBO nasal spray twice weekly, in addition to comprehensive SOC treatment (initial psychiatric hospitalization for a recommended ≥ 5 days and newly initiated or optimized oral antidepressant(s), per clinical judgement and practice guidelines) (Fig. 1). After the first dose, a one-time dose reduction to 56 mg ESK or PBO was allowed due to tolerability issues. Dose titrations/adjustment of SOC antidepressants occurred during the first 2 weeks of double-blind treatment. During the 9-week follow-up phase, patients continued SOC antidepressant treatment and discontinued ESK or PBO.

### Assessments

Depressive symptoms were assessed by the MADRS total score (range: 0–60) [22, 23]. Suicidality was assessed by the CGI-SS-r derived from the Suicide Ideation and Behavior Assessment Tool [24]. The CGI-SS-r (range: 0, normal, not at all suicidal to 6, among the most extremely suicidal patients) is a one-item, clinician-rated assessment of the current severity of a patient’s suicidal ideation and behavior. For both the MADRS and the CGI-SS-r scales, higher scores indicate worse symptomatology. Changes in MADRS total score and CGI-SS-r were assessed at 4 h and 24 h post first dose, twice per week pre-dose during the double-blind phase, and at varied time intervals during the follow-up phase (days 28–39: twice weekly; days 46–53: weekly; days 67–90: biweekly). Post dose assessment visits (ie, 4 h post dose on Day 1 and Day 25) were not included in the analysis performed for this publication because this post hoc analysis aimed to evaluate remission instead of rapid symptom reduction immediately after dosing.

### Time to remission and percent of days in remission

Two definitions of remission were evaluated, remission and consistent remission, that were defined as a MADRS total score ≤ 12 at any given visit and for two consecutive visits, respectively. Time to remission was calculated as the time (in days) between the randomization date and the first time the patient achieved remission. Time to consistent remission was calculated as the time (in days) between the randomization date and the date of the first of two consecutive visits where remission was achieved. For each definition of remission of MDD, a single criterion (i.e., MADRS total score ≤ 12) and a combined endpoint (i.e., MADRS total score ≤ 12 and CGI-SS-r ≤ 1 [not suicidal/questionably suicidal]) were examined. For consistent remission, both criteria (i.e., MADRS total score ≤ 12 and CGI-SS-r ≤ 1) had to be met for two consecutive visits. Patients were censored at the date of the last non-missing assessment if no event was identified over the entire study follow-up (days 1–90). Intermittent missingness was treated as a non-response. Monotone missingness was censored at the last non-missing assessment visit.

Number of days in remission (based on MADRS total score ≤ 12) was calculated for the double-blind treatment and follow-up phases. Percent of days in remission was calculated as the number of days in remission divided by the number of days in the study. When calculating the number of days in remission, the last observation carried backward was used within the double-blind treatment and post treatment follow-up phases separately (i.e., the last observed remission or non-remission status was carried backward until the visit with a non-missing value), due to the intermittent assessments at scheduled visits per protocol. For monotone missingness, values were not included in the number of days in the study.

### Statistical analysis

The analysis set included all randomized patients from ASPIRE I and ASPIRE II. The Kaplan–Meier product-limit method was used to estimate the median time (95% confidence interval [CI]) to remission of MDD from baseline. Univariate and multivariable Cox proportional hazards regression models were used to estimate hazard ratio (HR). The multivariable Cox hazards regression model included treatment and baseline score as covariates, and analysis center and SOC antidepressant treatment at baseline as stratification factors. For the number of days in remission, a Mann–Whitney U test was used to compare the median percent of days in remission between treatment arms. No adjustment for multiple comparisons was made.

## Results

A total of 451 (ESK + SOC: 226; PBO + SOC: 225) patients from the pooled studies were included in this analysis. Patient demographics and baseline characteristics were well balanced between the ESK + SOC and PBO + SOC groups (Table 1). Two patients in each treatment group were excluded from these analyses because they did not receive a dose of intranasal study drug after randomization. Notably, patients had a baseline mean MADRS total score of 40 (severe depression) and about 90% of patients were moderately to extremely suicidal.

**Table 1:** Baseline demographic and clinical characteristics

Characteristics	PBO + SOC (*N* = 225)	ESK + SOC (*N* = 226)
Age, mean (SD), years	39.6 (13.1)	40.5 (12.9)
Women, n (%)	140 (62.2)	134 (59.3)
MADRS total score^a^, mean (SD)	40.4 (6.0)	40.3 (5.6)
CGI-SS-r^a^, n (%)		
Questionably suicidal	6 (2.7)	6 (2.7)
Mildly suicidal	17 (7.6)	16 (7.1)
Moderately suicidal	61 (27.1)	64 (28.4)
Markedly suicidal	84 (37.3)	86 (38.2)
Severely suicidal	55 (24.4)	46 (20.4)
Extremely suicidal	2 (0.9)	7 (3.1)
Prior suicide attempt (lifetime)a, n (%)	140 (62.2)	144 (64.0)
Suicide attempt within the last month, n (%)	55 (24.4)	68 (30.1)
SOC AD monotherapy at baseline, n (%)	108 (48.0)	104 (46.0)
SOC AD augmentation therapy at baseline, n (%)	117 (52.0)	122 (54.0)

*AD* Antidepressant, *CGI-SS-r* Clinical Global Impression–Severity of Suicidality–revised, *ESK* Esketamine, *MADRS* Montgomery-Åsberg Depression Rating Scale, *PBO* Placebo, *SD* Standard deviation, *SOC* Standard of care

^a^*N* = 225 in ESK + SOC

### Time to remission of major depressive disorder

**Fig. 2:**
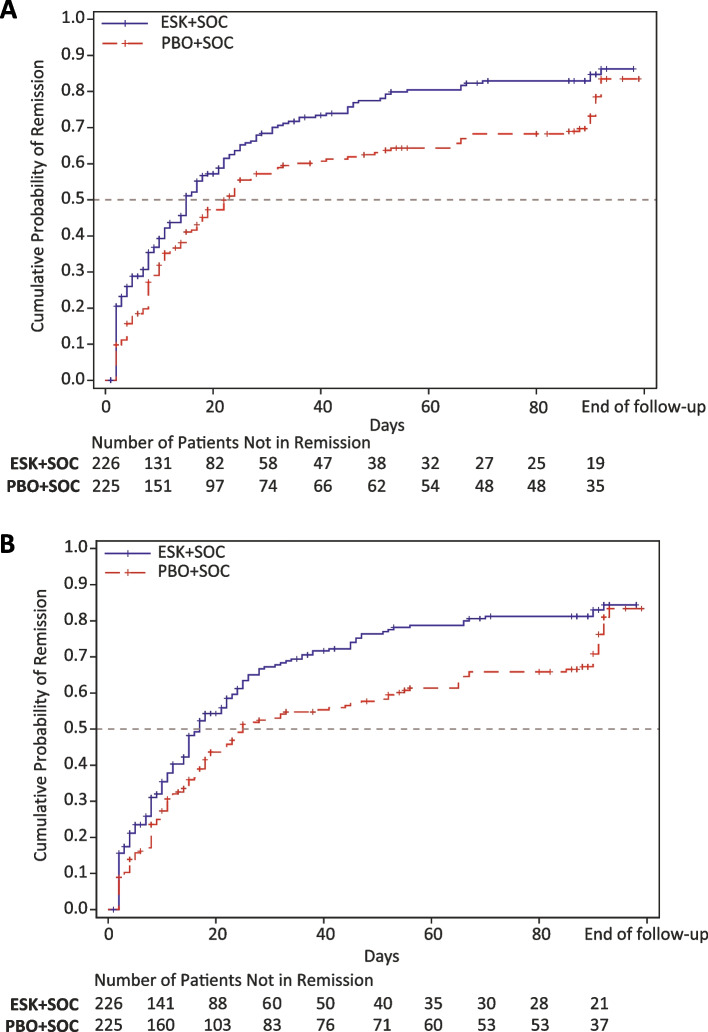
Kaplan–Meier curves of (**A**) time to remission of MDD based on MADRS and (**B**) time to remission of MDD based on the combined endpoint of MADRS and CGI-SS-r for the ESK + SOC and PBO + SOC groups. Cumulative probability of remission is the cumulative probability of achieving remission by criterion (A) or (B) by a given time. The numbers of patients at risk (ie, who have not yet remitted) for each of the groups are shown below the survival curves. CGI-SS-r, Clinical Global Impression-Severity of Suicidality-revised version; ESK, esketamine; MADRS, Montgomery-Åsberg Depression Rating Scale; PBO, placebo; SOC, standard of care

Time to remission of MDD (MADRS total score ≤ 12) was significantly shorter in patients treated with ESK + SOC versus PBO + SOC: median time, 15 versus 23 days; adjusted HR (95% CI), 1.47 (1.13, 1.92); *p* = 0.005 (Fig. 2A, Table 2). As shown in Fig. 2A, the ESK + SOC group had a higher cumulative probability of achieving remission at any given time compared to the PBO + SOC group. Similarly, time to achieving both MADRS total score ≤ 12 (remission of MDD) and CGI-SS-r ≤ 1 (not suicidal/questionably suicidal) was significantly shorter in the ESK + SOC group versus the PBO + SOC group: median time, 17 versus 25 days; adjusted HR (95% CI), 1.51 (1.15, 1.98); *p* = 0.003 (Fig. 2B, Table 2).

**Table 2:** Time to remission and consistent remission of MDD

	**ESK + SOC** **Median (95% CI)** ***N* = 226**	**PBO + SOC** **Median (95% CI)** ***N* = 225**	**Unadjusted HR (95% CI)**	***P*-value**	**Adjusted HR**^**a**^** (95% CI)**	***P*-value**
**Time to remission of MDD**						
**Using MADRS total score ≤ 12 as a single criterion**						
Days to remission	15 (12, 18)	23 (17, 28)	1.36 (1.10, 1.70)	0.006	1.47 (1.13, 1.92)	0.005
**Using MADRS total score ≤ 12 and CGI-SS-r ≤ 1 as a combined endpoint**						
Days to remission	17 (15, 22)	25 (19, 45)	1.39 (1.12, 1.74)	0.003	1.51 (1.15, 1.98)	0.003
**Time to consistent remission of MDD**						
**Using MADRS total score ≤ 12 as a single criterion**						
Days to remission	23 (21, 28)	50 (32, NE)	1.63 (1.27, 2.08)	0.0001	1.50 (1.12, 2.00)	0.007
**Using MADRS total score ≤ 12 and CGI-SS-r ≤ 1 as a combined endpoint**						
Days to remission	25 (22, 36)	52 (32, NE)	1.52 (1.18, 1.96)	0.001	1.42 (1.06, 1.91)	0.020

*CGI-SS-r* Clinical Global Impression–Severity of Suicidality–revised, *CI* Confidence interval, *ESK* Esketamine, *HR* Hazard ratio, *MADRS* Montgomery-Åsberg Depression Rating Scale, *MDD* Major depressive disorder, *NE* Not estimable, *PBO* Placebo, *SOC* Standard of care

^a^Adjusted for baseline score of individual measure, with analysis center and SOC antidepressant treatment as randomized stratification factors

Based on the single criterion of MADRS total score ≤ 12, the cumulative probability of remission was 65.2% in the ESK + SOC group versus 55.5% in the PBO + SOC group by day 25 (ie, end of the double-blind treatment phase); and 86.3% in the ESK + SOC group versus 83.5% in the PBO + SOC group by day 90 (ie, end of the follow-up phase) (*p* = 0.006). Based on the combined endpoint of MADRS total score ≤ 12 and CGI-SS-r ≤ 1, the cumulative probability of remission was 63.4% in the ESK + SOC group versus 51.3% in the PBO + SOC group by day 25; and 84.4% in the ESK + SOC group versus 83.4% in the PBO + SOC group by day 90 (*p* = 0.003).

### Time to consistent remission of major depressive disorder

**Fig. 3:**
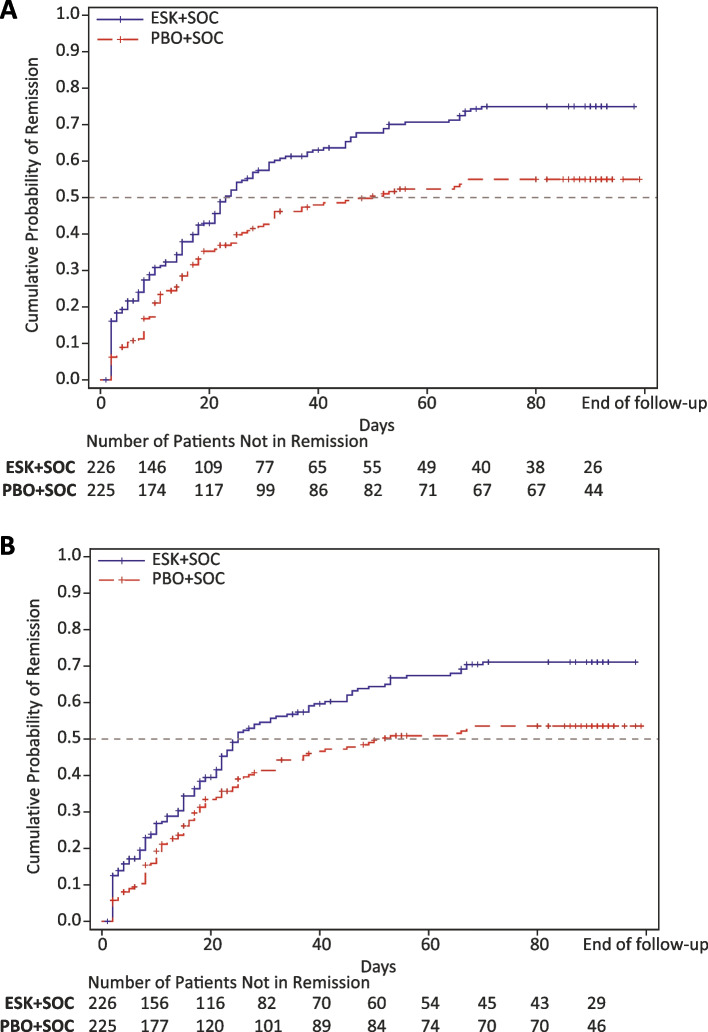
Kaplan–Meier curves of (**A**) time to consistent remission of MDD based on MADRS and (**B**) time to consistent remission of MDD based on the combined endpoint of MADRS and CGI-SS-r for the ESK + SOC and PBO + SOC groups. Cumulative probability of remission is the cumulative probability of achieving remission by criterion (A) or (B) by a given time. The numbers of patients at risk (ie, who have not yet remitted) for each of the groups are shown below the survival curves. CGI-SS-r, Clinical Global Impression-Severity of Suicidality-revised version; ESK, esketamine; MADRS, Montgomery-Åsberg Depression Rating Scale; PBO, placebo; SOC, standard of care

Time to consistent remission of MDD (MADRS total score ≤ 12 for two consecutive visits) was significantly shorter in patients treated with ESK + SOC versus PBO + SOC: median time, 23 versus 50 days; adjusted HR (95% CI), 1.50 (1.12, 2.00); *p* = 0.007 (Fig. 3A, Table 2). Time to achieving both MADRS total score ≤ 12 and CGI-SS-r ≤ 1 for two consecutive visits was significantly shorter in the ESK + SOC group versus the PBO + SOC group: median time, 25 versus 52 days; adjusted HR (95% CI), 1.42 (1.06, 1.91); *p* = 0.020 (Fig. 3B, Table 2).

Based on the single criterion of MADRS total score ≤ 12 for two consecutive visits, the cumulative probability of consistent remission was 54.2% in the ESK + SOC group versus 39.8% in the PBO + SOC group by day 25; and 75.0% in the ESK + SOC group versus 55.0% in the PBO + SOC group by day 90 (*p* = 0.0001). Based on the combined endpoint of MADRS total score ≤ 12 and CGI-SS-r ≤ 1 for two consecutive visits, the cumulative probability of consistent remission was 51.8% in the ESK + SOC group versus 39.0% in the PBO + SOC group by day 25; and 71.1% in the ESK + SOC group versus 53.5% in the PBO + SOC group by day 90 ( *p* = 0.001).

### Percent of days in remission of major depressive disorder

The percent of days in remission of MDD during the double-blind treatment phase in all randomized patients was significantly greater for the ESK + SOC group versus the PBO + SOC group: median percent of days in remission, 27.1% versus 8.3% (5 days versus 2 days during the 25 days of the double-blind treatment phase), *p* = 0.006 (Table 3). Similarly, among the patients who achieved remission (MADRS total score ≤ 12) during the double-blind treatment phase, the percent of days in remission across both phases of the trial was significantly greater for the ESK + SOC group versus the PBO + SOC group: median percent days in remission, 58.3% versus 41.7%, *p* = 0.037 (Table 3).

**Table 3:** Days in remission of MDD

	**ESK + SOC**	**PBO + SOC**	***P*-value**
**Days in remission**^**a**^** achieved during DB phase**	***N* = 224**	***N* = 225**	
Median days in remission, DB phase	5	2	
Median % of days in remission, DB phase	27.1	8.3	0.006
**Days in remission**^**a**^** for patients who achieved remission during DB phase**	***N* = 138**	***N* = 117**	
Median days in remission, DB + FU phases	13	10	
Median % days in remission, DB + FU phases	58.3	41.7	0.037
**Days in remission**^**a**^** achieved during FU phase**	***N* = 189**	***N* = 185**	
Median days in remission, FU phase	37	23	
Median % days in remission, FU phase	63.5	38.1	0.049
**Days in remission**^**a**^** for patients who achieved remission during FU phase**	***N* = 145**	***N* = 127**	
Median days in remission, FU phase	49	42	
Median % days in remission, FU phase	81.0	77.8	0.333

*DB* Double-blind, *ESK* Esketamine, *FU* Follow-up, *MDD* Major depressive disorder, *SOC* Standard of care

^a^Remission defined as MADRS total score ≤ 12

Furthermore, during the follow-up phase (when patients received SOC antidepressant treatment only), the percent of days in remission continued to be greater for the ESK + SOC group versus the PBO + SOC group: median percent of days in remission, 63.5% versus 38.1%, *p* = 0.049 (Table 3). Among the patients who achieved remission (MADRS total score ≤ 12) during the follow-up phase, the percent of days in remission was also greater for the ESK + SOC group versus the PBO + SOC group: median percent days in remission, 81.0% versus 77.8%, *p* = 0.333 (Table 3).

## Discussion

In ASPIRE I and II, ESK demonstrated a rapid reduction of depressive symptoms in patients with MDD with active suicidal ideation and intent who were experiencing a psychiatric emergency [16, 17]. Here, we evaluated clinically relevant and important endpoints in these same patients. The results of this post hoc analysis demonstrated that, compared to PBO + SOC, ESK + SOC treatment resulted in a higher proportion of patients who achieved remission and consistent remission of MDD, and significantly and consistently shortened the time to both remission and consistent remission. Similar results were obtained when a single criterion of remission (MADRS total score ≤ 12) was used as well as when a more stringent combined endpoint of remission incorporating depressive symptoms (MADRS total score ≤ 12) and severity of suicidality (CGI-SS-r ≤ 1) was used. Patients in the ESK + SOC group also spent a significantly greater percent of days in remission during the double-blind treatment and follow-up phases than patients in the PBO + SOC group.

In practice, temporary hospitalization leaves patients with MDD and suicidality vulnerable in the near term while antidepressant treatment takes weeks to be effective [10]. In addition, compared to patients with depression without suicidality, patients with depression and suicidality are less likely to improve or achieve remission with oral antidepressants [25]. Our results suggest that ESK may address the unmet needs of this population, providing rapid and sustained reduction of symptomatology and attainment of remission.

A large body of literature points to the importance of rapidly achieving remission in patients with MDD and suicidality [26]. Patients with MDD who achieve remission exhibit better functioning and an improved prognosis [27], improved health-related [28, 29] and “back-to-normal” [30] quality of life, and improved functional status [31]. The incidence of suicide is 21-fold lower during remission than during a major depressive episode [19]. American Psychiatric Association guidelines call for addressing the underlying disease (eg, depression) as the main treatment for suicidality [9]. In addition, improvement of depressive symptoms and remission are the most important attributes considered by physicians when assessing the readiness to discharge a patient with MDD and acute suicidal ideation with intent from the hospital [32].

In addition to the humanistic benefits of achieving remission, there are economic benefits as well. In patients with treatment-resistant depression, healthcare resource utilization (HRU) is lower during remission than during a major depressive episode [33]. Another study showed that patients with moderate or severe treatment-resistant depression have greater HRU and higher medical costs than those with a mild form of the illness [34]. Findings are similar in MDD — healthcare costs, HRU, and productivity losses are lower among patients who remit compared to those who do not achieve remission [31, 35, 36].

A strength of our analysis is that multiple, increasingly stringent assessments of remission were used and that the results obtained were robust and consistent. The benefits of ESK + SOC over PBO + SOC were maintained during the follow-up phase, when ESK treatment had been discontinued.

Limitations of this analysis include the post-hoc nature of the evaluation and the lack of adjustment for multiple comparisons. In addition, the suicidality endpoint (CGI-SS-r), while an important component of the remission assessment, did not show a statistically significant difference favoring ESK in the ASPIRE studies. Therefore, the results should be interpreted in line with the exploratory nature of the analysis. Finally, MADRS and CGI-SS-r assessments were performed intermittently, thus the remission analyses used values at each visit or two consecutive visits and days of remission in between two assessments were based on the calculation of last observation carried backward as described in the Methods.

## Conclusions

In summary, in this post hoc analysis of patients with MDD with suicidal ideation or behavior, treatment with ESK + SOC, compared to PBO + SOC, resulted in greater proportions of patients achieving remission and significantly shorter time to remission (using increasingly stringent definitions of remission incorporating depressive symptoms and severity of suicidality), as well as a significantly greater percent of time spent in remission. These findings highlight the clinical benefits of ESK treatment to address high unmet needs in adults with MDD with acute suicidality.

### Supplementary Information

**Additional file 1:** List of Institutional Review Boards and Independent Ethics Committees.

## Data Availability

The data sharing policy of Janssen Pharmaceutical Companies of Johnson & Johnson is available at: https://www.janssen.com/clinical-trials/transparency. Requests for access to the study data can be submitted through the Yale Open Data Access (YODA) Project site at: NCT03039192: https://yoda.yale.edu/nct03039192-double-blind-randomized-placebo-controlled-study-evaluate-efficacy-and-safety-intranasal NCT03097133: https://yoda.yale.edu/nct03097133-double-blind-randomized-placebo-controlled-study-evaluate-efficacy-and-safety-intranasal

## Abbreviations

AD: Antidepressant
CGI-SS-r: Clinical Global Impression-Severity of Suicidality-revised
CI: Confidence interval
DB: Double-blind
ESK: Esketamine
FU: Follow-up
HR: Hazard ratio
MADRS: Montgomery-Åsberg Depression Rating Scale
MDD: Major depressive disorder
NE: Not estimable
PBO: Placebo
SD: Standard deviation
SOC: Standard of care
