# Supplementary material for: Esketamine versus placebo on time to remission in major depressive disorder with acute suicidality
Source: BMC Psychiatry. 2023 Aug 11;23:587. doi: 10.1186/s12888-023-05017-y (PMC10416356; doi:10.1186/s12888-023-05017-y)
Supplement: Supplementary file 1 — Additional file 1: List of Institutional Review Boards and Independent Ethics Committees. [file 12888_2023_5017_MOESM1_ESM.pdf]

Additional File 1. List of Institutional Review Boards and Independent Ethics Committees

ARGENTINA*

Comité de Ética e Investigación del Sanatorio, Cordoba
Comite de Ética del Instituto Medico Platense CEDIMP, Buenos Aires
Comité de Ética en Investigación Clínica Privada de Salud Mental Stanta Teresa de Avila, Buenos Aires
Comité de Ética de CER Investigaciones Clínicas – CECIC, Buenos Aires
Comité de Ética en Investigacion Burzaco Comité de Etica en Investigacion Burzaco, Buenos Aires
Comité de Ética en Investigaciones FLENI, Buenos Aires

AUSTRIA

Ethikkkommission der Med. Universität Wien, und des Allgemeinen Krhs. der Stadt Wien, Wein
Kepler Universitätsklinikum Ethikkommission des Landes Oberösterreich, Linz

BELGIUM*

Ethisch Comité g UZ Gent, Gent
O.L.V. Ziekenhuis IRB, Aalst
Toetsingscommissie Ethiek GGZ Broeders van liefde, Bierbeek

BRAZIL*

National Committee of Ethics in Research (CONEP), Brasilia
Comite de Etica em Pesquisa da UFMG – COEP, Belo Horizonte
Comitê de Ética em Pesquisa do Investiga - Instituto De Pesquisas, Campinas
Comitê de Ética em Pesquisa da Faculdade de Medicina do ABC, Santo Andre
Comite de Ética em Pesquisa do Hospital Universitário, Salvador & Fortaleza
Comitê de Ética em Pesquisa do Hospital São Carlos, Fortaleza, Ceará
Comissao de Etica para analise de projetos de pequisa - CAPPesq-HCFMUSP, San Paulo

BULGARIA

Ethics Committee for Clinical Trials

CANADA

St. Michael’s Hospital Research Ethics Board, Toronto, Ontario

CZECH REPUBLIC*

Eticka komise Ustredni vojenske nemocnice Praha, Prague
Eticka komise IKEM a Thomayerovy nemocnice, Prague
Fakultni nemocnice Brno IRB-EC, Brno

ESTONIA

Tallinn Medical Research Ethics Committee

FRANCE

CPP Sud-Ouest et Outre-Mer III, Service de Pharmacologie Clinique, Leon

GERMANY

Ethikkommission der Medizinischen Fakultät der Albert-Ludwigs-Universität Freiburg

Ethik-Kommission des Fachbereichs Medizin der Johann Wolfgang Goethe-Universität

Landesamt für Gesundheit Und Soziales Berlin Geschäftsstelle der Ethik-Kommission des Landes Berlin

HUNGARY

Central Ethics Committee Medical Research Council Ethics Committee for Clinical Pharmacology

KOREA

Chonnam National University Hospital IRB
Samsung Medical Center IRB
Kyung Hee University Medical Center IRB
Seoul National University Hospital IRB
Korea University Ansan Hospital IRB

LITHUANIA

Lithuanian Bioethics Committee, Vilnius

MALAYSIA

Medical Research and Ethics Committee, Kompleks Institut Kesihatan Negara
Medical Research Ethics Committee, University Malaya Medical Centre

POLAND

Niezalezna Komisja Bioetyczna do Spraw Badan Naukowych przy Gdanskim
Uniwersytecie Medycznym, Gdansk

SOUTH AFRICA

Pharma Ethics

SPAIN

CEIC Hospital Universitari Vall d Hebron
Hospital de Navarra - Ceic de Navarra, Pamplona

TAIWAN

Institutional Review Board of Tri-Service General Hospital
Chung Shan Medical University Hospital IRB
Taipei Medical University Joint Institutional Review Board
Institutional Review Board, Taipei Veterans General Hospital

TURKEY

Uludag University Medical Faculty Clinical Research Ethics Committee, Bursa

UNITED STATES

Biomedical Research Alliance of New York IRB (Lake Success, NY)
Chesapeake IRB (Columbia, MD)
John Hopkins Medicine IRB (Baltimore, MD)
New York State Psychiatric Institutional Review Board (New York, NY)
Office of Research Integrity (Charleston, SC)
Rush University Medical Center Institutional Review Board (Chicago, IL)
Sharp HealthCare Institutional Review Board (San Diego, CA)
Springfield Committee for Research Involving Human Subjects (SCRIHS) (Springfield, IL)
Sterling Institutional Review Board (Atlanta, GA)
UCSD Human Subjects Research Protection Program (La Jolla, CA)
University at Buffalo Institutional Review Board (Buffalo, NY)
University of Connecticut School of Medicine IRB (Farmington, CT)
Univeristy of Louisville, Medical Center Institutional Review Board (Louisville, KY)
University of North Carolina at Chapel Hill - Office of Research Ethics, Chapel Hill, NC
UT Southwestern Medical Center Institutional Review Board (Dallas, TX)
Western Institutional Review Board (Puyallup, WA)

* Two or more Independent Ethics Committees approved the study protocol/amendments at sites in Argentina, Belgium, Brazil, and Czech Republic.
